# Catalysis-Based Fluorometric Method for Semiquantifying Trace Palladium in Sulfur-Containing Compounds and Ibuprofen

**DOI:** 10.1021/acs.joc.4c00651

**Published:** 2024-05-28

**Authors:** Judey T. DaRos, Miho Naruse, Jasmyne M. Mendoza, Abhimanyu Nangunoori, Jacob H. Smith, Jill E. Millstone, Kazunori Koide

**Affiliations:** Department of Chemistry, University of Pittsburgh, 219 Parkman Avenue, Pittsburgh, Pennsylvania 15260, United States

## Abstract

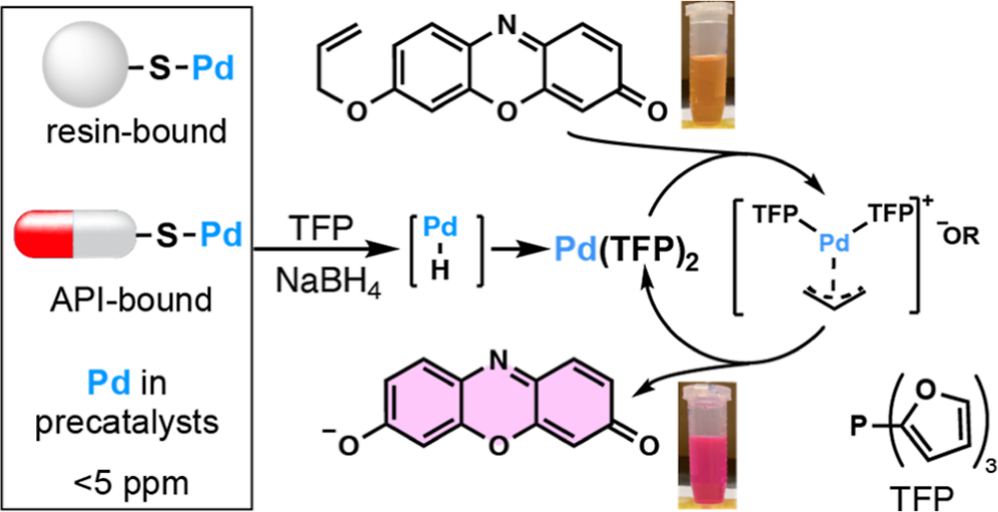

Trace palladium in synthetic materials can be rapidly and inexpensively semiquantified by a catalysis-based fluorometric method that converts resorufin allyl ether to resorufin. However, whether sulfur compounds would interfere with this method has not been systematically studied. Herein, we show that although thiourea in solution interferes with quantification, sulfide, thiol, and thiocarbamate do not. The fluorometric method can also detect palladium bound to sulfur-based scavenger resin and outperform inductively coupled plasma mass spectrometry for detecting trace palladium in ibuprofen.

## Introduction

Palladium is presumably the most frequently used transition metal in organic synthesis today.^1,2^ Palladium is also toxic, requiring rigorous metal removal in active pharmaceutical ingredients (APIs).^3−7^ However, universally effective protocols to remove palladium do not exist because individual APIs bind palladium differently. Researchers typically screen dozens of techniques to lower palladium contents and quantify palladium by inductively coupled plasma optical emission spectrometry (ICP-OES) or inductively coupled plasma mass spectrometry (ICP-MS). Because these quantification methods are not high-throughput, the metal removal process can take weeks. For example, in a case study by process and analytical chemists at Bristol Myers Squibb, the typical workflow required 8–10 weeks to assess and remove palladium after a palladium-catalyzed reaction was performed.^5^ During the screening phase, a rapid and semiquantitative method would accelerate the identification of suitable metal-scavenging methods. Palladium contamination is also well-recognized in medicinal chemistry.^8^

**Figure 1:**
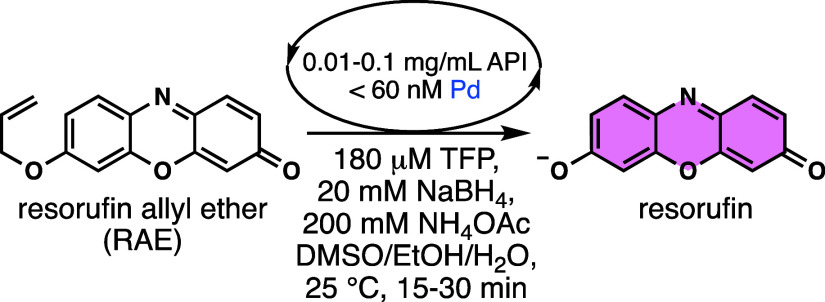
Fluorogenic conversion of RAE to resorufin. TFP = tri(2-furyl)phosphine; API = active pharmaceutical ingredient.

To expedite the semiquantification of palladium in APIs, we previously reported catalysis-based fluorometric/colorimetric technology (Figure 1)^9−17^ and its kinetic study.^18^ Resorufin allyl ether (RAE) is nonfluorescent, and palladium catalysis with tri(2-furyl)phosphine (TFP) and NaBH_4_ can cleave the allylic ether to form a magenta-fluorescent molecule, resorufin. The most robust procedure requires NaBH_4_,^15^ without which sulfur-containing compounds interfere with the deallylation reaction.^16^ The samples do not need to be digested by acid when NaBH_4_ is used, unlike ICP-OES and ICP-MS techniques.^12,15^ Additionally, the fluorometric method can be employed with lower API quantities (0.01–0.1 mg/mL) than ICP-OES and ICP-MS (>1 mg/mL) and has been implemented at Merck Research Laboratories.^12,13^ The Tolnai group also used our earlier method to detect trace palladium in synthetic amine prepared by a palladium-catalyzed reaction.^19^

Nonetheless, skepticism persists in the literature. The concerns include “the majority of the fluorescence-based approaches to palladium detection do not work effectively when other metals are present in significant amounts” and “the presence of base in the analyte solution can pose a challenge as can variations in the parameters of the Tsuji-Trost reaction used to generate the fluorescent species in the visualization reaction”.^20^ We have previously shown that 11.5 equiv of other metals do not interfere with the method (see Figure 6 of our previous publication^15^). Prior publications also show that amines do not interfere with the semiquantitative fluorometric method.^12,15−17^ This is presumably because the concentrations of an API and TFP in the assay solutions are both in the upper micromolar range, and TFP should bind more favorably than amines to palladium. Additionally, aliphatic amines are protonated at pH 7 and highly solvated in water, mitigating palladium chelation. Optimization of the method in the presence of 200 mM NH_4_OAc further negates the presence of amines at micromolar concentrations. However, we previously found that sulfur compounds interfered with the detection and quantification of palladium^16^ except when NaBH_4_ was used.^15^ With limited examples in prior studies, it remained unclear which sulfur-containing functional groups could interfere with the fluorometric method.

Finally, although we previously validated our fluorometric method through comparison with palladium concentrations measured by ICP-MS, the method’s sensitivity has not been investigated. This article shows that our fluorometric method is equally or more sensitive than ICP-MS to detect or quantify palladium in APIs and is compatible with sulfide, thiol, and thiocarbamate.

## Results and Discussion

**Figure 2:**
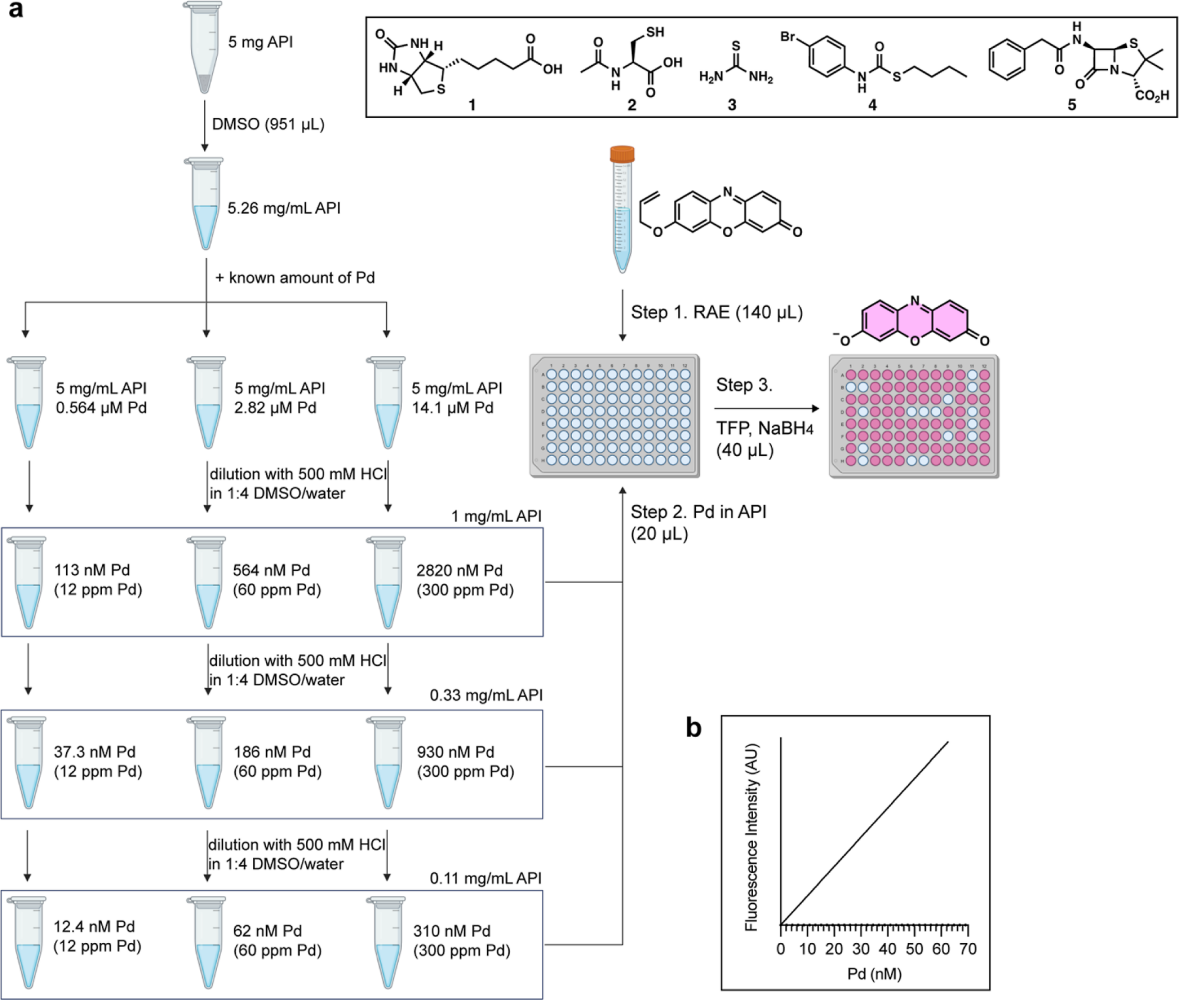
Flowchart of the palladium detection assay. (a) API models **1**–**5** (biotin, *N*-acetyl-l-cysteine, thiourea, thiocarbamate, and penicillin) were spiked with known amounts of palladium corresponding to 12, 60, and 300 ppm in the solid state and diluted to a range of palladium concentrations to be used in the assay. (b) Theoretical calibration curve for palladium with 60 μM RAE, 180 μM TFP, 200 mM NH_4_OAc, and 20 mM NaBH_4_ in 4.4:18:77.6 v/v/v DMSO/H_2_O/EtOH. FI was measured on a microplate reader (λ_ex_ = 525 nm, λ_em_ = 580–640 nm) immediately (*t*_0_) and 15 min later (*t*_15_). FI (arbitrary unit) = (slope) × [Pd (nM)].

We first asked which sulfur-containing functional groups might be detrimental to the fluorometric method. To this end, sulfur-containing compounds **1**, **2**, **3**, **4**, and **5** (Figure 2a) as API models were dissolved in DMSO and spiked with various known amounts of palladium. These solutions were serially diluted so that the final palladium concentrations were within the linear range of the calibration curve and tested using our method 2 and 4 days after preparing the palladium-spiked samples. Each black 96-well plate was treated with a solution of RAE (step 1), palladium-spiked API solutions (step 2), and a solution of TFP and NaBH_4_ (step 3). Fluorescence intensities (FIs) were measured at 0 and 15 min after step 3. A calibration curve (Figure 2b) was generated following the same procedure using a commercially available palladium standard solution serially diluted to the final concentrations of 0–62.5 nM palladium in each well. To process the data, we subtracted the FI of the wells at 0 min from the FIs of the calibration curve and API solutions at 15 min. A line-of-best-fit equation was used to interpolate expected FIs (=100% signal recovery) based on the known amount of palladium in the spiked API solutions (Figures S1 and S2). The signal recovery was then calculated using the ratio of FIs from API solutions over expected FIs (Tables S9 and S10).

The results are summarized in Table 1. Remarkably, sulfides **1** and **5**, thiol **2**, and thiocarbamate **4** did not interfere with the semiquantification of palladium in the samples. The majority of the % signal recovery in these respective APIs was within 70–150%, which is an acceptable accuracy range according to USP 38th, chapter 233. Palladium in the presence of thiourea **3** could be detected with 31–36% signal recovery only when the palladium content was as high as 300 ppm in solid thiourea. These data show the scopes and limitations of the fluorometric semiquantitative method. As we reported previously, palladium in the presence of biotin could not be detected in the absence of NaBH_4_.^16^ Therefore, although a Pd–S bond is generally considered strong (197 ± 6 kJ mol^–1^), a Pd–H bond is stronger (208 ± 9 kJ mol^–1^), overcoming sulfur-related interference in three of the four sulfur-containing functional groups. Thiourea binds palladium through the Pd–S bond formation,^21^ but bulky thiourea derivatives do not inhibit palladium catalysis,^22^ indicating that thiourea-mediated interference may depend on substituents.

**Table 1:** Percent Signal Recovery of Palladium-Spiked APIs Determined from Experimental and Expected Fluorescence Intensities^a^

theoretical Pd (ppm) in solid API	12	60	12	60	300	60
Pd in assay solution (nM)	3.76	6.26	11.2	18.8	31.3	56.3
API (mg/mL) in the spiked sample	0.33	0.11	1	0.33	0.11	1
2 Days Post API Spiking						
1 biotin	145 ± 15	155 ± 27	164 ± 17	131 ± 10	99 ± 27	89 ± 7
**2** *N*-acetyl-l-cysteine	113 ± 14	123 ± 7	102 ± 1	115 ± 9	101 ± 5	70 ± 4
**3** thiourea	0	0	0	11 ± 4	31 ± 1	0
**4** thiocarbamate	129 ± 18	100 ± 4	148 ± 17	69 ± 9	86 ± 10	58 ± 1
**5** penicillin	107 ± 8	123 ± 8	64 ± 2	117 ± 4	104 ± 9	85 ± 6
4 Days Post API Spiking						
**1** biotin	69 ± 2	124 ± 11	123 ± 17	106 ± 4	77 ± 5	65 ± 4
**2** *N*-acetyl-l-cysteine	42 ± 6	93 ± 5	43 ± 1	78 ± 2	78 ± 3	49 ± 2
**3** thiourea	0	30 ± 8	0	10 ± 1	36 ± 6	1 ± 1
**4** thiocarbamate	93 ± 10	38 ± 5	95 ± 4	24 ± 5	35 ± 4	21 ± 1
**5** penicillin	78 ± 6	111 ± 14	65 ± 8	89 ± 7	86 ± 6	61 ± 4

a Data are the mean ± SD, tested in three replicates. A simple linear regression was performed to obtain a line of best-fit equations (Figures S1 and S2) from which expected FIs at corresponding palladium concentrations were interpolated. To determine % signal recovery, the ratio of observed FIs for palladium-spiked API solutions to expected FIs was taken and multiplied by 100 (Tables S9 and S10). Detailed calculation is described in Supporting Information.

**Figure 3:**
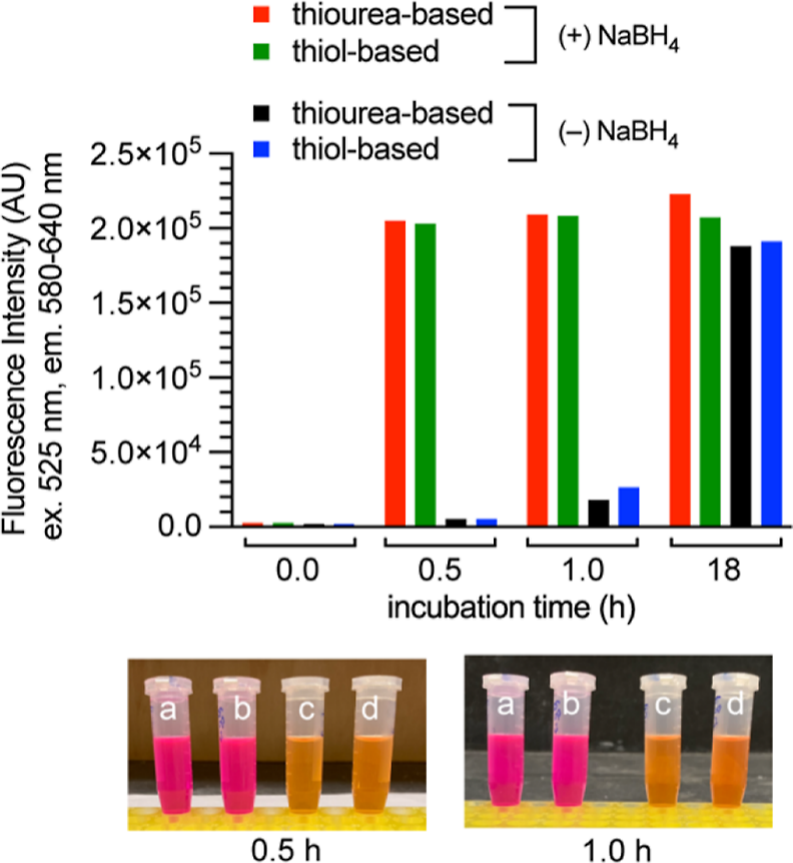
Catalytically inactive palladium is detected rapidly in the presence of NaBH_4_ and catalyzes the conversion of RAE (yellow) to resorufin (magenta). The resin was loaded with Pd(OAc)_2_ and washed three times with EtOH. (a) Thiourea-based resin with NaBH_4_. (b) Thiol-based resin with NaBH_4_. (c) Thiourea-based resin without NaBH_4_. (d) Thiol-based resin without NaBH_4_. Conditions: 60 μM RAE, 180 μM TFP, 202 mM NH_4_OAc, and 20 or 0 mM NaBH_4_ in 2.4:78.6:10 v/v/v DMSO/EtOH/water. Incubation at room temperature. The suspended resin was 6.8 mg for thiol-based resin and 4.3 mg for thiourea-based resin.

Our second question was whether our method could be used to monitor the presence or absence of palladium in sulfur-based resin. Trace palladium in APIs is frequently removed using thiourea- or thiol-based resins. Some of these resins are expensive, and recycling can reduce costs. Therefore, it is useful to determine whether palladium remains bound after washing the resin. As such, we incubated thiol- and thiourea-based resins with a solution of Pd(OAc)_2_ in DMSO, washed the resin three times with EtOH, and treated the resulting resins with a solution of RAE and TFP in DMSO/EtOH/water with and without NaBH_4_. As Figure 3 shows, the presence of NaBH_4_ enabled the detection of resin-bound palladium after 30 min. Without NaBH_4_, the FI was not comparable until 18 h later. Therefore, our technology can detect thiourea- and thiol-bound palladium on resins, and the use of NaBH_4_ significantly enhances the sensitivity. The successful detection of thiourea-bound palladium on the resin may be because, unlike in solutions (Table 1), the number of palladium-bound thiourea molecules is limited due to restriction by the polymer scaffold, leaving the palladium loosely bound.

**Figure 4:**
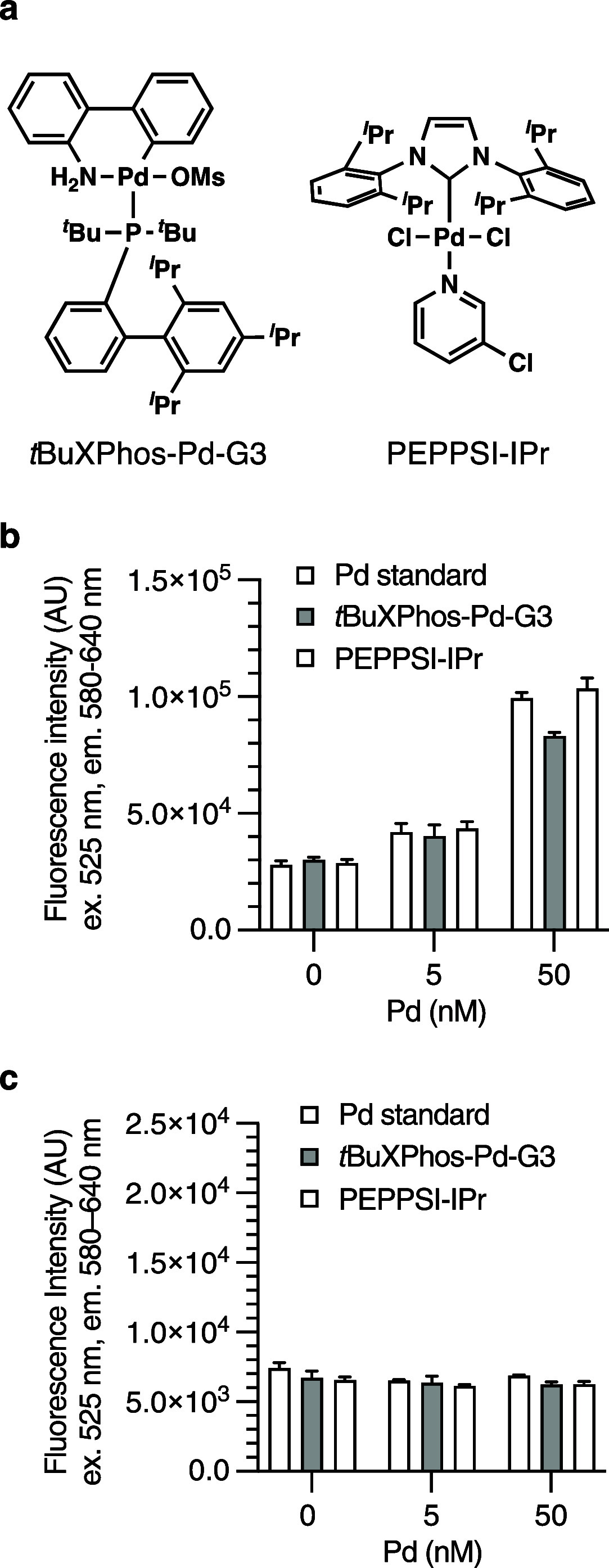
Comparison of palladium standard solution, *t*BuXPhos-Pd-G3, and PEPPSI-IPr for their catalytic activity (Tables S12 and S13). FIs were compared using a paired *t* test. Data are the mean ± SEM. Tested in three replicates. The incubation time was 15 min data at *t*_15_ are shown. (a) Structures of *t*BuXPhos-Pd-G3 and PEPPSI-IPr. (b) No significant difference was seen between palladium standard and *t*BuXPhos-Pd-G3 (ns, *p* = 0.335), as well as between palladium standard and PEPPSI-IPr (ns, *p* = 0.115). Conditions: 60 μM RAE, 180 μM TFP, 200 mM NH_4_OAc, 75 mM NaOH, and 20 mM NaBH_4_ in 7.4:77.6:15 v/v/v DMSO/EtOH/water. (c) Without NaBH_4_, the conversion of RAE to resorufin does not occur. Conditions: 60 μM RAE, 180 μM TFP, 200 mM NH_4_OAc, 75 mM NaOH, and 0 mM NaBH_4_ in 7:78:15 v/v/v DMSO/EtOH/water. Incubation at room temperature.

The third unanswered question was whether palladium species in resting states in APIs can catalyze the O-deallylation of RAE under the assay conditions. We chose the Buchwald precatalysts *t*BuXPhos-Pd-G3 and PEPPSI-IPr (Figure 4a) as model reagents.^23^Figure 4b indicates that *t*BuXPhos-Pd-G3 and PEPPSI-IPr are as catalytically active as the palladium standard solution under the indicated reaction conditions. Palladium species in resting states are not readily available, and this study is thus limited to these two precatalysts; however, this work implies that the affinities of hydride and TFP toward palladium can activate palladium species in resting states. Figure 4c shows that NaBH_4_ is essential for robust palladium detection using our fluorometric method.

The last question is about the sensitivity of the fluorometric method. The technology does not need to be as accurate and precise as ICP-MS because it is meant to be an efficient screening tool to assess the metal removal efficiency. Given the real-world concerns about trace palladium in ibuprofen,^24,25^ we asked how low of a palladium concentration in the drug could be estimated. Previous work showed that 1 ppm palladium in APIs could be semiquantified.^11,12,15^ In this study, we spiked known amounts of *t*BuXPhos-Pd-G3 to solutions of ibuprofen and evaporated the solvents to prepare ibuprofen samples contaminated with palladium at 80, 20, 5, and 0.2 ppb. Using the standard addition technique, we attempted to quantify the palladium in the ibuprofen samples. As Table 2 shows, 80 ppb palladium in ibuprofen could be semiquantified with 21% error, while ≤20 ppb palladium could not be reliably quantified. The limit of quantification of routine ICP-MS analysis of palladium in organic materials is about 5 ppm and can be as low as 0.1–1 ppm if extensive sample preparation is performed.^26−28^ The current fluorometric method is, therefore, higher-throughput and less expensive to semiquantify the same or lower concentrations of palladium in ibuprofen.

**Table 2:** Theoretical (Actual) and Experimental Values for Trace Palladium Quantification Using Standard Addition^a^

actual Pd (ppb)	measured Pd (ppb)
80	97 ± 24
20	69 ± 19
5	91 ± 20
0.2	78 ± 29

a Data are the mean ± SD. 80 ppb was tested in four replicates, and other concentrations were tested in three replicates. The incubation time was 30 min. Conditions: 57 μM RAE, 171 μM TFP, 19 mM NaBH_4_, 190 mM NH_4_OAc, 71 mM NaOH, and 19 mM NaBH_4_ in 16.3:74.2:9.5 v/v/v DMSO/EtOH/water. Incubation at room temperature.

**Figure 5:**
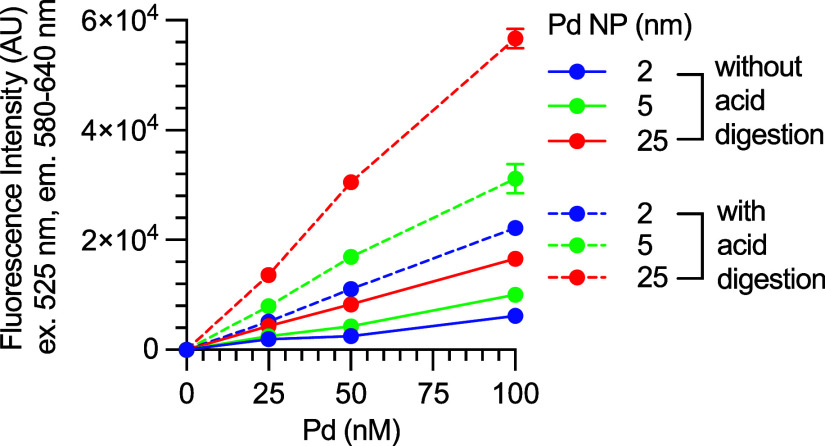
Determining the relative quantities of palladium in nanoparticles with the fluorometric method. Three palladium nanoparticles with average sizes of 2, 5, and 25 nm were treated with water or 20% aqua regia and diluted with 1:4 DMSO/water with 500 mM HCl. FI was measured immediately (*t*_0_) and 15 min later (*t*_15_). Data are the mean ± SEM, tested in three replicates. Conditions: 60 μM RAE, 180 μM TFP, 200 mM NH_4_OAc, 75 mM NaOH, and 20 mM NaBH_4_ in 7.4:77.6:15 v/v/v DMSO/EtOH/water. Incubation at room temperature.

Trace palladium in APIs may be in the form of palladium nanoparticles.^29^ As such, we prepared 2, 5, and 25 nm palladium nanoparticles according to the literature using 1 kDa poly(ethylene glycol)methyl ether thiol as a particle-terminating ligand.^30,31^ Each sample was treated with either water or 20% aqua regia in water. These solutions were then diluted with 500 mM HCl in 1:4 DMSO/water (the same procedure as experiments for Table 1) and subjected to the fluorometric method. As Figure 5 shows, the nondigested samples showed lower signals than those of the aqua regia-treated samples. However, it is important to note that the signals for each nanoparticle sample are mostly linearly correlated with the palladium concentrations. Therefore, although the fluorometric method is less quantitative without aqua regia digestion, it is still a valuable screening tool to rank samples for relative amounts of palladium after an API sample is treated with dozens of purification methods to prioritize the scavenging technique.

In summary, we show that the fluorometric/colorimetric method based on the conversion of RAE to resorufin is semiquantitative even in the presence of sulfide, thiol, and thiocarbamate but not in the presence of thiourea. The method’s semiquantitative nature and low material requirement (0.01–0.1 mg/mL) make it a valuable tool for screening palladium removal techniques. The method could detect palladium bound to thiol- and thiourea-based scavenger resins. We also show that the trace palladium species in API may not need to be catalytically active because NaBH_4_ and TFP activate them in the assay solution. Finally, the limit of semiquantification of palladium in ibuprofen is comparable to or lower than that of ICP-MS. Therefore, despite the concerns raised in the literature, the current fluorometric method is robust and sensitive.

## Data Availability

The data underlying this study are available in the published article and its Supporting Information.

## Supporting Information Available

Experimental procedures and raw data for figures in the main text (PDF)
