# Supplementary material for: Catalysis-Based Fluorometric Method for Semiquantifying Trace Palladium in Sulfur-Containing Compounds and Ibuprofen
Source: J Org Chem. 2024 May 28;89(11):8005–10. doi: 10.1021/acs.joc.4c00651 (PMC11165445; doi:10.1021/acs.joc.4c00651)
Supplement: Supplementary file 1 — jo4c00651_si_001.pdf [file jo4c00651_si_001.pdf]

Supporting Information

for

Catalysis-Based Fluorometric Method for Semiquantifying Trace Palladium in Sulfur-Containing Compounds and Ibuprofen

Judey T. DaRos, Miho Naruse, Jasmyne M. Mendoza, Abhimanyu Nangunoori, Jacob H. Smith, Jill E. Millstone, and Kazunori Koide*

Department of Chemistry, University of Pittsburgh

219 Parkman Avenue, Pittsburgh, Pennsylvania 15260, United States

koide@pitt.edu

Table of Contents

Instrumentation S3

Reagents S3

Supplementary Information on Procedures

- General temperatures S3
- Preparation of stored stock solutions S3
- General procedure for assay solutions S4
- General procedure for calibration curve S4
- Procedure for investigating sulfur compound compatibility (Table 1) S4
- **Table S1.** Sample calculation of ppm in solid state. Calculated from API and palladium concentrations in solution state. S5
- **Table S2:** Demonstrating the ppm corresponding to nM palladium in solution S5
- Procedure for detecting resin-bound palladium (Figure 3) S5
- Procedure for comparing catalytic activity of *t*BuXPhos-Pd-G3, PEPPSI^{TM}-IPr catalyst, and palladium standard solution (Figure 4) S6
- Procedure for quantifying palladium in ibuprofen by standard addition (Table 2) S7
- Procedure for palladium nanoparticle synthesis and characterization S7
- Procedure for investigating compatibility with palladium nanoparticles (Figure 5) S9

Raw Data and Supplementary Figures

- Detecting palladium in spiked API solutions (Table 1) S10
- Detecting resin-bound palladium (Figure 3) S15
- Comparison of palladium standard solution, *t*BuXPhos-Pd-G3, and PEPPSI^{TM}-IPr for their catalytic activity (Figure 4) S15
- Quantification of palladium by standard addition method (Table 2) S16

Investigation of compatibility with palladium nanoparticles (Figure 5) S20
Cited References S23

Instrumentation

Fluorescence measurements (λ_{ex} = 525 nm, λ_{em} = 580–640 nm) were carried out using black 96-well plates on a Modulus II Microplate Reader (Turner Biosystems) or a GloMax Discover microplate reader (Promega).

Reagents

Water in this study was purified by a Barnstead Nanopure Diamond Lab Water System. DMSO was used without purification. EtOH was USP-grade 200 proof. MeOH was HPLC-grade. A 1 mg/mL (1000 ppm = 9.4 mM) palladium standard solution in 10% HCl (2.7 M HCl) for atomic absorption spectroscopy (CAS#7440-30-5, catalog #196171000) was purchased from Fisher and stored at 24 °C. $NaBH_4$ pellets (CAS# 16940-66-2, catalog# 448495000, Acros Organics) were used for this study. 10 M NaOH aqueous solution (CAS# 1310-73-2, catalog# S255, Fisher Chemical), tris(2-furyl)phosphine (CAS#5518-52-5, catalog#T1643, TCI chemicals), D-(+) biotin (CAS# 58-85-5, catalog# AAA14207, Alfa Aesar), *N*-acetyl-L-cysteine (CAS# 616-91-1, catalog# TCA0905, TCI chemicals), thiourea (CAS# 62-56-6, catalog# AC220052500, Acros Organics), *S*-butyl *N*-(4-bromophenyl)thiocarbamate (CAS# 108540-54-1, catalog# S595802, Sigma-Aldrich), penicillin G sodium salt (CAS#69-57-8, catalog#AAJ6303206, Thermo Scientific Chemicals), PEPPSI^{TM}-IPr catalyst (CAS# 905459-27-0, $C_{32}H_{40}Cl_3N_3Pd$, catalog# 669032, Sigma-Aldrich), *t*BuXPhos-Pd-G3 (CAS #: 1447963-75-8, $C_{42}H_{58}NO_3PPdS$, catalog# 762289, Sigma-Aldrich), Deloxan MP metal scavengers (thio-functionalized polysiloxane) (catalog# 14-871, Strem Chemicals), and Deloxan THP II microporous thiourea-functionalized polysiloxane (catalog# 14-870, Strem Chemicals) were purchased from commercial sources and used without further purification. Potassium tetrachloropalladate (CAS#10025-98-6, catalog#205796), palladium acetylacetonate (CAS#14024-61-4, catalog#209015), oleylamine technical grade 70% (CAS#209015, catalog# O7805), sodium borohydride (CAS#16940-66-2, catalog#452882, Sigma-Aldrich), tetraoctylammonium bromide (CAS#14866-33-2, catalog#294136, Sigma-Aldrich), borane *tert*-butylamine complex (CAS#7337-45-3, catalog#180211), 1,2-hexadecanediol (CAS#6920-24-7, catalog#213748), and poly(ethylene glycol)methyl ether thiol (PEGSH) (MW = 1kDa) (CAS#134874-49-0, catalog#6161-1-07A, Synquest Laboratories) for nanoparticle synthesis were purchased from Sigma-Aldrich unless specified otherwise. Resorufin allyl ether was synthesized according to reported procedure.[1]

Supplementary Information on Procedures

General temperatures

All experiments were performed at 24–25 °C unless stated otherwise.

Preparation of stored stock solutions

Preparation of 5 mM RAE in DMSO

Resorufin allyl ether (5.0 mg) was dissolved in DMSO (3.8 mL) in an amber vial and stored at -20 °C.

Preparation of 250 ppm BHT in DMSO

BHT (5.0 mg) was dissolved in DMSO (18.2 mL), and the resulting solution was stored at 24 °C in an amber vial.

Preparation of 15.0 mM tris(2-furyl)phosphine (TFP) in DMSO containing 250 ppm BHT

TFP (70 mg) was dissolved in DMSO containing 250 ppm BHT (20.1 mL) in an amber vial, and the resulting solution was stored at 24 °C.

Preparation of 500 mM ammonium acetate (NH_4OAc) in 200-proof ethanol (EtOH)

Ammonium acetate (7.71 g) was dissolved in EtOH (200 mL), and the resulting solution was stored at 24 °C.

Preparation of 625 mM aqueous HCl

A solution of 11.65 M trace metal grade HCl (16.1 mL) was diluted in water (283.9 mL) in an amber bottle, and the resulting solution was stored at 24 °C.

*Preparation of 500 mM HCl in 1:4 v/v DMSO/water (**Solution A**)*

A solution of 625 mM aqueous HCl (100 mL) was diluted in DMSO (25 mL) in an amber bottle, and the resulting solution was stored at 24 °C.

Preparation of 2.0 mM palladium standard solution in 10% w/v HCl

A solution of 34-37% trace metal grade HCl (281.7 µL) was diluted to 10% w/v HCl in water (718.3 µL). A solution of 1000 ppm (9.4 mM) palladium standard solution in 10% w/v HCl (425 µL) was diluted with 10% w/v HCl (1.57 mL) to the final concentration of 2.0 mM palladium. The resulting solution was stored at 24 °C.

General procedure for assay solutions

Preparation of 85.7 µM RAE/286 mM NH_4OAc in 1.7:98.3 v/v DMSO/EtOH

The 5 mM RAE solution (0.32 mL) in DMSO was diluted with EtOH (1.68 mL) to obtain 800 µM RAE in 4:21 v/v DMSO/EtOH. This solution (1.61 mL) was then mixed with 500 mM NH_4OAc (8.58 mL) and EtOH (4.81 mL).

Preparation of 900 µM TFP/100 mM $NaBH_4$/375 mM NaOH in 3:22:25 v/v/v DMSO/EtOH/water

One $NaBH_4$ pellet (1.00 g) was crushed and dissolved in cold aqueous 10 M NaOH (9.91 mL) to obtain 2.67 M $NaBH_4$ and 10 M NaOH in water. This solution (450 µL) was diluted in water (2.55 mL) to obtain 400 mM $NaBH_4$ and 1.5 M NaOH in water. All solutions containing $NaBH_4$ were kept on ice until added to reaction mixtures. The 400 mM $NaBH_4$ solution (750 µL) was mixed with EtOH (1.32 mL), 15 mM TFP in DMSO containing 250 ppm BHT (180 µL), and water (750 µL) to obtain 900 µM TFP/100 mM $NaBH_4$/375 mM NaOH in 3:22:25 v/v DMSO/EtOH/water.

General procedure for calibration curve

Generating calibration curve using palladium standard solution

A 2.0 mM palladium standard solution in 10% trace metal grade HCl (100 µL) was diluted with **Solution A** (900 µL) to prepare 200 and 20 µM palladium. The 20 µM palladium solution (1.00 mL) was then transferred to a deep 96-well plate. Two-fold serial dilutions using **Solution A** produced 10000, 5000, 2500, 1250, 625, 313, 156, 78.1, 39.1, and 19.6 nM palladium solutions. The final concentrations of palladium in the assay wells were 62.5, 31.3, 15.6, 7.81, 3.91, 1.96, and 0 nM.

Procedure for investigating sulfur compound compatibility (Table 1)

Spiking palladium in API samples

Biotin (**1**), *N*-acetyl-L-cysteine (**2**), thiourea (**3**), *S*-butyl *N*-(4-bromophenyl)thiocarbamate (**4**), and penicillin (**5**) (5.26 mg each) were dissolved in DMSO (1.00 mL) to prepare 5.26 mg/mL API solutions. The 2.0 mM palladium standard solution in 10% w/v HCl (70.5 µL) was diluted with **Solution A** (429.5 µL) to prepare 282 µM palladium (500 µL). Two rounds of 5-fold dilutions with **Solution A** produced 56.4 and 11.3 µM palladium solutions. The 5.26 mg/mL API samples (285 µL) were spiked with 282 µM, 56.3 µM, 11.3 µM palladium, **Solution A** (15 µL each) to make 5 mg/mL API with 14.1 µM, 2.82 µM, 564 nM, and 0 nM palladium corresponding to 300, 60, and 12 ppm (Table S1). The 5 mg/mL API solutions (90 µL) were diluted with **Solution A** (360 µL) to prepare 1.0 mg/mL API solutions. Two rounds of 3-fold dilutions were performed to prepare solutions with concentrations for use in assay (Table S2).

Table S1. Sample calculation of ppm in solid state. Calculated from API and palladium concentrations in solution state.

API (mg/mL)	Pd (µM)	Volume (µL)	API (mg)	Pd (nmols)	Pd (ng)	ppm in solid state (ng Pd/mg API)
5	2.82	300	1.5	0.846	90	60

Table S2: Demonstrating the ppm corresponding to nM palladium in solution.

ppm in solid state (ng Pd/mg API)	[Pd] in 5 mg/mL API	[Pd] in 1 mg/mL API	[Pd] in 0.33 mg/mL API	[Pd] in 0.11 mg/mL API
12	563.8 nM	112 nM	37.6 nM	12.5 nM
60	2.82 µM	563 nM	188 nM	62.6 nM
300	14.1 µM	2820 nM	940 nM	313 nM

Assay

Final conditions: 60 µM RAE, 180 µM TFP, 200 mM NH_4OAc, 75 mM NaOH, 20 mM $NaBH_4$ in 4.4:18:77.6 v/v/v DMSO/water/EtOH.

A solution of 85.7 µM RAE/286 mM NH_4OAc in 1.7:98.3 v/v DMSO/EtOH (140 µL) was transferred to wells in a black 96-well plate. Solutions of 1, 0.33, and 0.11 mg/mL API with varying palladium concentrations (20 µL) were subsequently added. To generate a calibration curve, solutions of 625–0 nM palladium (20 µL) were added to separate wells to generate a calibration curve. Finally, the solution of 900 µM TFP/100 mM $NaBH_4$/375 mM NaOH in 3:22:25 v/v/v DMSO/EtOH/water (40 µL) was added to all wells. Fluorescence values were measured immediately after the addition of the $NaBH_4$-TFP solution (0 min) and after incubating at room temperature away from light for 15 min. Calibration curves were generated each day. Data for calibration curves shown in Tables S3 and S4 were used in the analysis of data collected on the same days in Tables S5, S6, S7, S9, and S10.

Procedure for detecting resin-bound palladium (Figure 3)

Loading $Pd(OAc)_2$ on metal scavengers

Deloxan MP metal scavengers (1.2 mmol/g thio-functionalized polysiloxane; 56 mg, 67 µmol) or 1.2 mmol/g Deloxan THP II microporous thiourea-functionalized polysiloxane (56 mg, 67 µmol) was added to $Pd(OAc)_2$ (10 mg, 45 µmol) followed by DMSO (1.78 mL). These mixtures were incubated at 25 °C for 15 min. The solids were filtered, separated from liquids, washed with EtOH (2 mL × 3), and dried under an air atmosphere.

Preparation of 240 µM RAE/400 mM NH_4OAc in 4.8:95.2 v/v DMSO/EtOH

Solutions of 5 mM RAE in DMSO (72 µL), 500 mM NH_4OAc in EtOH (1200 µL), and EtOH (228 µL) were combined in a 2-mL microcentrifuge tube.

Preparation of 400 mM $NaBH_4$/1.5 M NaOH in water

A $NaBH_4$ pellet (1.00 g) was dissolved in ice-cold 10 M NaOH (9.9 mL) to prepare 2.67 M $NaBH_4$. A fraction of the resulting solution (450 µL) was diluted with water (2.55 mL).

Preparation of 0 or 80 mM $NaBH_4$, 720 µM TFP in 4.8:25.2:70 v/v/v DMSO/EtOH/water

Solutions of 400 mM $NaBH_4$ in 1.5 M NaOH (600 µL), 15 mM TFP in DMSO (144 µL), water (1500 µL), and EtOH (756 µL) were combined. To prepare 0 mM $NaBH_4$ solution, 1.5 M NaOH (600 µL) was added instead of 400 mM $NaBH_4$.

Assay

Final conditions: 60 μM RAE, 180 μM TFP, 202 mM NH_4OAc and 20 or 0 mM $NaBH_4$ in 2.4:78.6:10 v/v/v DMSO/EtOH/water.

The palladium-loaded thiol-based resin (6.8 mg) or palladium-loaded thiourea-based resin (4.3 mg) was suspended in EtOH (2.00 mL) in a 5-mL conical tube. This solution was treated with the solution of 240 μM RAE (1.00 mL) and the solution of 80 mM $NaBH_4$ and 720 μM TFP (1 mL) or the solution of 0 mM $NaBH_4$ and 720 μM TFP (1 mL). Fractions (200 μL) of these resulting solutions were transferred to a black 96-well plate in one replicate to measure the fluorescence values immediately after the addition of the $NaBH_4$-TFP solution (0 min), after incubating at room temperature for 0.5, 1, and 18 h.

Procedure for comparing catalytic activity of *t*BuXPhos-Pd-G3, PEPPSI^{TM}-IPr, and palladium standard solution (Figure 4)

Preparation of solutions with palladium pre-catalysts

PEPPSI^{TM}-IPr catalyst (18.7 mg) and *t*BuXPhos-Pd-G3 (12.4 mg) were each dissolved in DMSO (13.8 mL and 7.81 mL, respectively) to the final concentration of 2.00 mM palladium. A 2-fold serial dilution was performed on each 2.00 mM palladium solution in 1:1 v/v DMSO/water to obtain 1000 and 500 μM palladium solutions. Ten-fold serial dilutions on the 500 μM palladium solutions produced 50 μM, 5 μM, 500 nM, and 50 nM palladium solutions. The solution of 1:1 v/v DMSO/water was used as 0 nM palladium solution in the assay.

Preparation of solutions with palladium standard solution

A 2-fold serial dilution was performed on 625 mM aqueous HCl in 1:1 v/v DMSO/water to obtain 313 mM HCl. This solution was used to dilute 2.0 mM palladium standard solution to 1000, 500, and 5 μM, 500 and 50 nM palladium. The solution of 313 mM HCl was used as a 0 nM palladium standard solution in the assay.

Preparation of 900 μM TFP/375 mM NaOH in 3:22:25 v/v/v DMSO/EtOH/water

A solution of 10 M NaOH (112 μL) was diluted with water (1.388 mL) to obtain 750 mM NaOH in water. EtOH (880 μL), 15 mM TFP in BHT in DMSO (120 μL), and 750 mM NaOH in water (1.00 mL) were mixed to obtain 900 μM TFP/375 mM NaOH in 3:22:25 v/v/v DMSO/EtOH/water.

Assay with 20 mM $NaBH_4$

Final conditions: 0, 5, 50 nM palladium, 180 μM TFP, 60 μM RAE, 20 mM $NaBH_4$, 75 mM NaOH, 200 mM NH_4OAc in 7.4:15:77.6 v/v/v DMSO/water/EtOH. The final well volume was 200 μL tested in three replicates.

A solution of 85.7 μM RAE/286 mM NH_4OAc in 1.7:98.3 v/v DMSO/EtOH (140 μL) was transferred to wells in a black, 96-well plate. Solutions of 0, 5, and 50 nM palladium standard, *t*BuXPhos-Pd-G3, and PEPPSI^{TM}-Ipr catalyst (20 μL) were transferred to wells. A multi-channel pipette was used to transfer 900 μM TFP/100 mM $NaBH_4$/375 mM NaOH in 3:22:25 v/v/v DMSO/EtOH/water (40 μL) to all wells. Fluorescence values were measured on the microplate reader immediately after the addition of the $NaBH_4$-TFP solution (0 min) and after incubating at room temperature away from light for 15 min.

Assay with 0 mM $NaBH_4$

Final conditions: 0, 5, 50 nM palladium, 180 μM TFP, 60 μM RAE, 0 mM $NaBH_4$, 75 mM NaOH, 200 mM NH_4OAc in 7:78:15 v/v/v DMSO/EtOH/water. The final well volume was 200 μL tested in three replicates.

A solution of 85.7 μM RAE/286 mM NH_4OAc in 2.6/148.4 v/v/v DMSO/EtOH (140 μL) was transferred to wells in a black, 96-well plate. Solutions of 0, 5, and 50 nM palladium standard, *t*BuXPhos-Pd-G3, and PEPPSI-*i*Pr catalyst (20 μL) were transferred to wells. Solutions of 0, 5, and 50 nM palladium standard, *t*BuXPhos-Pd-G3, and PEPPSI^{TM}-IPr catalyst (20 μL) were transferred to wells. A multi-channel pipette was used to transfer 900 μM TFP/375 mM NaOH in 3:22:25 v/v/v DMSO/EtOH/water (40 μL) to all wells.

Fluorescence values were measured on the microplate reader immediately after the addition of the $NaBH_4$-TFP solution (0 min) and after incubating at room temperature away from light for 15 min.

Procedure for quantifying palladium in ibuprofen by standard addition (Table 2)

Preparation of 80, 20, 5, and 0.2 ppb palladium in ibuprofen

In a 2-dram vial, ibuprofen (200 mg) was dissolved in MeOH (500 µL). The API sample was spiked to contain 25 ppm palladium by addition of 500 µg/mL *t*BuXPhos-Pd-G3 solution in DMSO (74.6 µL) to the ibuprofen in MeOH (500 µL). After a 1 h incubation, the spiked ibuprofen solution was placed on a rotary evaporator to remove MeOH and then lyophilized overnight. This sample (80 mg) was dissolved in DMSO (2 mL) using a volumetric flask to obtain 9400 nM palladium in DMSO (1 µg/mL palladium). The diluent was prepared by dissolving ibuprofen (1.000 g) in DMSO (25 mL) to obtain 40 mg/mL ibuprofen. The 9400 nM palladium solution (1.00 mL) was then diluted in 40 mg/mL ibuprofen (1.50 mL) to prepare 3760 nM palladium. This solution was then diluted five-fold three times to obtain 30.1 nM palladium. This solution was then diluted four-fold twice to obtain 7.525 and 1.88 nM palladium. The 1.88 nM solution was diluted five-fold twice to obtain 0.0752 nM palladium. These solutions correspond to 80, 20, 5, and 0.2 ppb (ng palladium/g ibuprofen) in solid state respectively.

Calculation

For 200 mg of ibuprofen, 5000 ng of palladium is needed to prepare an ibuprofen sample containing 25 ppm palladium. A solution of 500 µg/mL *t*BuXPhos-Pd-G3 (794.37 g/mol) is equivalent to 66.98 µg/mL palladium. Spiking 66.98 µg/mL palladium (74.6 µL) in the solution containing ibuprofen (200 mg) will result in 25 ppm in solid state.

Preparation of 840, 420, and 210 nM palladium spike solutions

A 2.52 mM solution of *t*BuXPhos-Pd-G3 was prepared by dissolving *t*BuXPhos-Pd-G3 (12.1 mg) in DMSO (6.05 mL). This solution was then serially diluted in DMSO to 2.52 µM, 840, 420, and 210 nM palladium.

Preparation of 375 mM NaOH in 3:22:25 v/v/v DMSO/EtOH/water for reaction blanks

A 3:22:25 v/v/v DMSO/EtOH/water solution was prepared. This solution (3.85 mL) was then used to dilute 10 N NaOH (150 µL) to 375 mM NaOH.

Assay

Final conditions: 171 µM TFP, 57 µM RAE, 19 mM $NaBH_4$, 71 mM NaOH, 190 mM NH_4OAc, 0, 10, 20, or 40 nM palladium, 3.81 mg/mL ibuprofen, 16.3:74.2:9.5 v/v/v DMSO/EtOH/H_2O. The final well volume was 210 µL tested in two or three replicates.

A solution of 85.7 µM RAE/286 mM NH_4OAc in 1.7:98.3 v/v DMSO/EtOH (140 µL) was added to wells in a black 96-well plate. A solution of 80, 20, 5, or 0.2 ppb palladium in 40 mg/mL ibuprofen (20 µL) was added to wells. Wells were spiked with 840, 420, or 210 nM palladium solutions (10 µL) for a final concentration of 40, 20, or 10 nM palladium. A solution of 375 mM NaOH in 3:22:25 v/v/v DMSO/EtOH/water (40 µL) was added to wells designated as reaction blanks. A solution of 900 µM TFP/100 mM $NaBH_4$/375 mM NaOH in 3:22:25 v/v/v DMSO/EtOH/ water (40 µL) was added to wells not designated as reaction blanks. All outer wells were filled with water (200 µL). Fluorescence values were measured on the microplate reader immediately after the addition of the $NaBH_4$-TFP solution (0 min) and after incubating at room temperature away from light for 30 min.

Procedure for palladium nanoparticle synthesis and characterization

Synthesis of 2 nm palladium nanoparticles (Pd NPs)

2-nm Pd NPs were synthesized using a procedure described by Zou et al.[2] Briefly, K_2PdCl_4 (0.4 mmol) was dissolved in water (12 mL) in a 20-mL scintillation vial. In a separate vial, tetraoctylammonium bromide

(TOAB) (2 mmol) was dissolved in toluene (25 mL). The clear TOAB solution was then layered on top of the aqueous K_2PdCl_4 solution. The biphasic mixture was vortexed until the organic layer became red and the aqueous layer became clear. The organic phase containing the $(TOA)PdCl_4$ complex was removed and transferred to a 250-mL round-bottom flask equipped with a stir bar, followed by the addition of oleylamine (OAm) (1.264 mL). $NaBH_4$ (8 mmol) dissolved in water (2 mL) was then added with vigorous stirring and allowed to sit for 1 h before the particles were precipitated with EtOH and isolated via centrifugation, followed by resuspension in $CHCl_3$.

Synthesis of 5 nm Pd NPs

5-nm Pd NPs were synthesized using a procedure described by Mazumder et al.[3] Briefly, $Pd(acac)_2$ (0.1 mmol) was dissolved in OAm (5 mL) and heated to 70 °C under argon. At this temperature, a separate, room temperature solution of borane tert-butylamine complex (100 mg) in OAm (2.5 mL) was injected into the heated reaction flask. The reaction was then heated to 90 °C and held at 90 °C for 1 h. After cooling to room temperature, particles were precipitated with EtOH and isolated via centrifugation, followed by resuspension in $CHCl_3$.

Synthesis of 25nm Pd NPs

25-nm Pd NPs were synthesized using a method developed by the Millstone group. The synthetic procedure was completed using standard air-free techniques. $Pd(acac)_2$ (0.1 mmol) was dissolved in OAm (1 mL). Separately, OAm (9 mL) and HDD (1 mmol) were added to a round bottom flask equipped with a stir bar and condenser. This mixture was then degassed at 100 °C for 1 h. The solution was then heated to 250 °C under argon, at which point the solution of $Pd(acac)_2$ was injected and held at 250 °C for 3 h. After cooling to room temperature, particles were precipitated with EtOH and isolated via centrifugation, followed by resuspension in $CHCl_3$.

Ligand Exchange and Aqueous Phase Transfer of Pd NPs with 1kDa PEGSH

Pd NPs of each size were phase transferred into water by ligand exchange with 1kDa PEGSH. In all cases, purified OAm capped Pd NPs were dispersed in $CHCl_3$ (5 mL). A solution of 20 mM 1kDa PEGSH in $CHCl_3$ (5 mL) was then added to the NP solutions, and the mixture was left stirring for 24 h. After incubation, the PEGSH-functionalized Pd NPs were precipitated with the addition of hexanes and isolated via centrifugation. The supernatant was removed, and the resulting pellet was resuspended in water (5 mL). The Pd content of these solutions was then measured by inductively coupled plasma-optical emission spectrometry (ICP-OES) and diluted with water to obtain 1 mM stock solutions.

Inductively Coupled Plasma Optical Emission Spectrometry (ICP-OES)

ICP-OES analysis was performed using an argon flow with a PerkinElmer, Inc. Optima spectrometer (Department of Chemistry, University of Pittsburgh). An ultrapure aqua regia solution was prepared with a 3:1 ratio of hydrochloric acid (Sigma-Aldrich, > 99.999% trace metal basis) and nitric acid (Sigma-Aldrich, > 99.999% trace metal basis) and diluted with water for a 5% v/v aqua regia matrix. Unknown Pd concentrations were determined by comparison to a 7-point standard curve with a range of 0.10–10 ppm of Pd (0.10, 0.50, 1.0, 2.5, 5.0, 7.5, and 10 ppm) prepared by volume using a Pd standard for ICP (Fluka, TraceCERT 1000 ± 2 mg/L Pd in HCl), diluted in a 5% aqua regia matrix. All standards and unknown samples were measured 3 times and averaged.

High-Resolution Transmission Electron Microscopy (HRTEM)

Pd NP samples were prepared for TEM by drop-casting an aliquot of the purified solution in water onto carbon film-coated copper TEM grids (Ted Pella, Inc., Redding, CA) for bright field imaging. TEM characterization for all Pd NPs was performed on a Hitachi H9500 Environmental TEM with an accelerating

voltage of 300 kV (Nanoscale Fabrication and Characterization Facility, Petersen Institute of Nanoscience and Engineering, Pittsburgh, PA). The size distributions of the NPs were determined by measuring 250 NPs from various areas of the grid using ImageJ 1.53k (National Institutes of Health, USA) (Figure S4)

Procedure for investigating compatibility with palladium nanoparticles (Figure 5)

Preparation of nanoparticle samples

Palladium nanoparticles of 2 nm, 5 nm, and 25 nm in water (1 mM palladium determined by ICP-OES) were diluted to 500 µM palladium using water or 20% aqua regia. Aqua regia was prepared by mixing a 3:1 ratio of 34-37% trace metal grade HCl and 67-70% trace metal grade nitric acid and diluted with water to make 20% aqua regia. The 500 µM palladium solutions of Pd NPs were subsequently diluted using Solution A to make 50 µM, 5 µM, 1 µM, 500 nM, and 250 nM palladium solutions. As a negative control, water (1 mL) was also subjected to the addition of 5 or 20% aqua regia (1 mL) and subsequent dilution with Solution A. As a positive control, the 2.0 mM palladium standard solution in 10% trace metal grade HCl was diluted with water to make a 1.0 mM palladium standard solution. This solution was diluted with water, 5% aqua regia, or 20% aqua regia to make a 500 µM palladium solution. These standard solutions were subsequently diluted using Solution A to make 50 µM, 5 µM, and 500 nM palladium solutions (Figure S5).

Assay

Final Conditions: 60 µM RAE, 180 µM TFP, 200 mM NH_4OAc, 75 mM NaOH, 20 mM $NaBH_4$ in 4.4:18:77.6 v/v/v DMSO/water/EtOH. The final well volume was 200 µL tested in three replicates.

A solution of 85.7 µM RAE/286 mM NH_4OAc in 2.6/148.4 v/v DMSO/EtOH (140 µL) was transferred to wells in a black, 96-well plate. Solutions of 2-nm, 5-nm, and 25-nm Pd NPs of 1 µM, 500 nM, and 250 nM palladium diluted with water or 20% aqua regia (20 µL) were transferred to wells. Standard solutions of 0 or 500 nM palladium diluted with water, 5% aqua regia, or 20% aqua regia (20 µL) were transferred to wells. A multi-channel pipette was used to transfer 900 µM TFP/375 mM NaOH in 3:22:25 v/v/v DMSO/EtOH/water (40 µL) to all wells. Fluorescence values were measured on the microplate reader immediately after the addition of the $NaBH_4$-TFPsolution (0 min) and after incubating at room temperature away from light for 15 min.

Raw Data and Supplementary Figures

Detecting palladium in spiked API solutions (Table 1)

Table S3. Raw fluorescence values used to generate calibration curves in Figure S1. Calibration curve solutions were made fresh for each day of testing. The remaining fluorescence intensity of the 0 nM palladium calibration solution was also subtracted from calibration solutions, forcing the calibration curve through the origin to simplify analysis and account for experimental variation.

2 days post spiking APIs												
Pd (nM)	t_0			t_{15}			t_{15}-t_0			(t_{15}-t_0) - 5815		
0	8280	7526	8338	14756	12310	14524	6476	4784	6186	661	-1031	371
1.96	8461	7914	9050	16967	14343	19173	8506	6429	10123	2690	614	4308
3.91	9552	8957	9399	23749	18931	22180	14198	9974	12781	8382	4159	6966
7.81	11793	10420	9953	34793	28377	29447	23000	17957	19494	17184	12141	13679
15.6	11641	11971	11976	43270	35271	41559	31629	23300	29584	25813	17485	23768
31.3	16728	18058	16326	66623	67293	66330	49895	49235	50004	44079	43419	44189
62.5	24192	25069	22405	101518	99079	99604	77326	74009	77199	71511	68194	71384
4 days post spiking APIs												
Pd (nM)	t_0			t_{15}			t_{15}-t_0			(t_{15}-t_0) - 6388		
0	8620	7968	8149	16371	14096	13436	7750	6127	5287	1362	-261	-1101
1.96	9064	9500	9645	19006	19914	19397	9942	10414	9752	3554	4026	3363
3.91	10048	10767	10223	24098	26813	23593	14050	16045	13371	7661	9657	6982
7.81	11919	10849	11094	33561	30453	30995	21642	19605	19902	15254	13217	13513
15.6	13094	11987	12982	45139	41181	44454	32045	29194	31472	25657	22806	25084
31.3	19203	19198	20112	77635	72694	76177	58432	53495	56065	52044	47107	49677
62.5	30751	28597	29491	121132	104976	111260	90381	76379	81769	83993	69991	75381

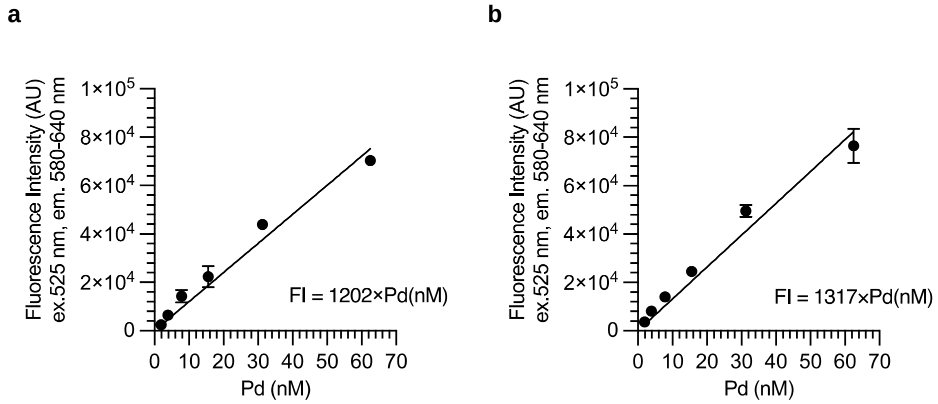

Figure S1. Calibration curves generated from Table S3. A simple linear regression was performed to generate respective equations. Equation was used to determine expected fluorescence values of APIs (biotin, N-acetyl-L-cysteine, thiourea, and thiocarbamate) 2 (a) and 4 (b) days after spiking.

Table S4. Raw fluorescence values used to generate calibration curves in Figure S2. Penicillin was tested 2 days and 4 days post spiking, and calibration curve solutions were made fresh for each day of testing.

2 days post spiking APIs

Pd (nM)	t_0			t_{15}			t_{15}-t_0			(t_{15}-t_0) - 2302		
0	7818	7919	7790	10384	10330	9718	2566	2411	1928	264	109	-374
1.96	8287	8573	8634	11876	13186	12781	3589	4613	4147	1287	2311	1845
3.91	8741	8900	8724	15352	16830	16648	6611	7930	7924	4309	5628	5622
7.81	9798	10603	10204	23277	24019	22733	13479	13416	12529	11177	11114	10227
15.6	11765	11242	12092	34460	39743	36532	22695	28501	24440	20393	26199	22138
31.3	18364	17597	14561	61807	62488	53420	43443	44891	38859	41141	42589	36557
62.5	25901	23226	17377	104178	80969	80547	78277	57743	63170	75975	55441	60868

4 days post spiking APIs

Pd (nM)	t_0		t_{15}		t_{15}-t_0		(t_{15}-t_0) - 2886	
0	7620	7612	10723	10280	3104	2668	218	-218
1.96	6878	7983	14570	14709	7692	6725	4806	3839
3.91	7965	8328	19274	19046	11309	10718	8423	7832
7.81	8901	9377	25519	28883	16618	19506	13732	16620
15.6	10211	9794	36090	43793	25879	33999	22993	31113
31.3	14581	15870	61686	64157	47105	48287	44219	45401
62.5	17935	24948	95768	98962	77832	74014	74946	71128

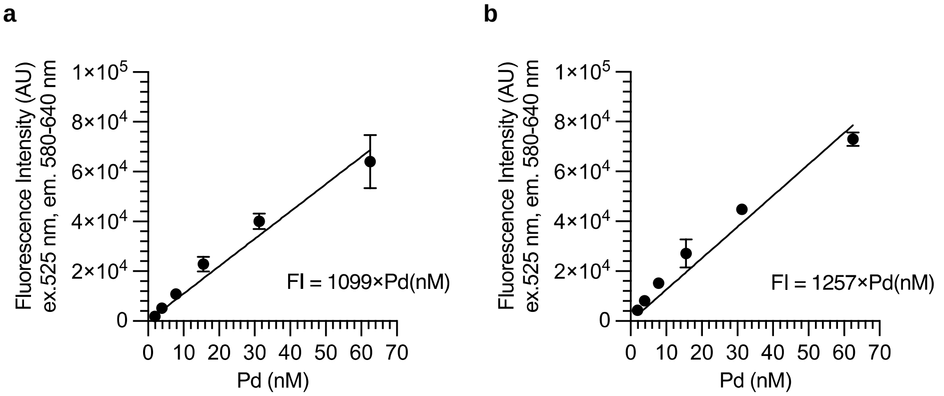

Figure S2. Calibration curves generated from Table S4. A simple linear regression was performed to generate respective line-of-best-fit equations. Equation was used to determine expected fluorescence values of API (penicillin) 2 (a) and 4 (b) days after spiking.

Table S5. Raw fluorescence values for APIs (biotin, N-acetyl-L-cysteine, thiourea, and thiocarbamate) tested 2 days post spike. Data processing is described in Table S3 caption.

2 days post spiking APIs		Biotin			N-Acetyl-cys			Thiourea			Thiocarbamate		
Pd (nM)	API (mg/mL)	t_0	t_{15}	(t_{15}- t_0) - 5815	t_0	t_{15}	(t_{15}- t_0) - 5815	t_0	t_{15}	(t_{15}- t_0) - 5815	t_0	t_{15}	(t_{15}- t_0) - 5815
3.76	0.33	9049	20950	6086	9204	20489	5470	7521	10649	-2687	10163	21746	5768
		9251	21360	6294	9762	21007	5430	7704	11812	-1707	9585	20505	5105
		9201	22326	7310	8941	19121	4365	7506	12001	-1320	9598	22097	6684
6.26	0.11	10752	26073	9506	9753	24393	8825	8005	13447	-373	11264	24577	7498
		10854	30159	13490	9652	24619	9152	8416	14246	15	11856	25533	7862
		10859	28712	12038	10302	25963	9846	8397	13455	-757	11226	24361	7320
11.2	1	12788	40878	22275	9862	29346	13669	6663	9623	-2855	14596	42589	22178
		12399	37895	19681	9600	29057	13642	6635	9131	-3319	14539	40175	19821
		12790	42968	24363	8825	28465	13825	6363	11045	-1133	13131	36598	17652
18.8	0.33	13375	46227	27037	12948	46926	28163	8091	15722	1816	11212	32396	15369
		13794	50784	31175	13921	45490	25754	7863	16063	2385	12365	35906	17726
		14203	50356	30338	12051	41977	24111	8045	17327	3467	12043	31345	13487
31.3	0.11	19849	70186	44522	15403	58452	37234	9323	26924	11786	21679	62613	35119
		15100	46312	25397	16227	58658	36616	10762	27813	11236	21557	61007	33635
		18100	65273	41358	14708	60795	40272	10673	28034	11546	20067	53889	28007
56.3	1	24466	68041	37760	16254	67409	45340	6734	4456	-8093	21400	66082	38867
		24600	69052	38637	15454	66804	45534	6618	3056	-9377	19830	65319	39674
		24976	60916	30125	17209	73681	50657	6704	3916	-8603	18222	63019	38982

Table S6. Raw fluorescence values for APIs (biotin, N-acetyl-L-cysteine, thiourea, and thiocarbamate) tested 4 days post spike. Data processing is described in Table S3 caption.

4 days post spiking APIs		Biotin			N-Acetyl-cys			Thiourea			Thiocarbamate		
Pd (nM)	API (mg/mL)	t_0	t_{15}	(t_{15}- t_0) - 6388	t_0	t_{15}	(t_{15}- t_0) - 6388	t_0	t_{15}	(t_{15}- t_0) - 6388	t_0	t_{15}	(t_{15}- t_0) - 6388
3.76	0.33	9191	18901	3322	9421	17994	2185	7789	10856	-3321	9790	20284	4106
		9483	19247	3376	9890	18581	2303	7689	10630	-3447	9806	20811	4617
		9050	18968	3530	9339	17425	1698	7699	10658	-3429	12416	23920	5116
6.26	0.11	11382	28642	10872	10809	24519	7322	8979	17527	2160	9276	18912	3248
		10689	26283	9206	11119	25068	7561	8843	18398	3167	9849	19678	3441
		11121	28118	10609	10772	25239	8079	9083	17456	1985	9239	18216	2589
11.2	1	11925	38467	20154	9649	22364	6327	7477	11174	-2691	11683	32103	14032
		11147	36450	18915	9735	22656	6533	7266	11251	-2403	12199	33211	14624
		10958	32615	15269	9720	22435	6327	7467	11910	-1945	11002	30890	13500
18.8	0.33	15478	47867	26001	15153	41283	19742	8305	17092	2399	11074	24597	7135
		14852	48357	27117	16680	42501	19433	8851	18046	2807	10039	22752	6325
		15030	46708	25290	15763	41055	18904	8155	16610	2067	10205	21163	4570
31.3	0.11	18510	58366	33468	17561	56876	32927	13735	35497	15374	13701	32889	12800
		17992	53986	29606	17366	54543	30789	14658	38453	17407	13175	34845	15282
		17282	56139	32469	17152	56191	32651	9826	28569	12355	13449	35488	15651
56.3	1	17973	69298	44937	17928	60840	36524	7755	15586	1443	11586	34089	16115
		19019	75518	50111	20387	65006	38231	7987	14816	441	12760	34790	15642
		20040	77051	50623	18748	60185	35049	7956	15101	757	12608	33887	14891

Table S7. Raw fluorescence values for penicillin tested 2 and 4 days post spike. Data processing is described in Table S3.

Penicillin		2 days post spiking APIs			4 days post spiking APIs		
Pd (nM)	API (mg/mL)	t_0	t_{15}	$(t_{15}- t_0)$ - 2302	t_0	t_{15}	$(t_{15}- t_0)$ - 2886
3.76	0.33	8043	14646	4301	7931	14191	3373
		8148	15256	4806	7647	14322	3789
		7931	14393	4160	7459	14306	3961
6.26	0.11	9435	19598	7861	8999	19336	7451
		8958	19843	8583	8961	21381	9534
		8837	20033	8894	9069	21180	9225
11.2	1	8911	19024	7811	8680	21876	10310
		8159	18180	7719	7394	19479	9199
		8510	18954	8142	8145	19050	8018
18.8	0.33	10243	37519	24974	9897	33493	20710
		9722	35226	23202	10079	32379	19414
		9552	36388	24534	10273	36073	22914
31.3	0.11	13746	48362	32314	16938	52029	32206
		12035	51200	36863	12279	47634	32469
		12076	52354	37976	12152	51538	36500
56.3	1	12297	69936	55337	10511	58779	45382
		15702	66257	48253	9105	55212	43222
		12191	67962	53469	17672	60984	40425

Table S8. Sample signal recovery calculation used to generate values in Table 1, S9, and S10.

2 days post spiking APIs											
Theoretical Pd (ppm) in solid API	Biotin (mg/mL)	Pd (nM)	Expected FI= 1202×Pd(nM)	t_0	t_{15}	$t_{15}- t_0$	Observed $(t_{15}- t_0)$ - 5815	Observed / Expected	% Signal Recovery	Mean	SD
300	0.11	31.3	37623	19849	70186	50337	44522	1.18	118	99	27
				15100	46312	31212	25397	0.68	68		
				18100	65273	47173	41358	1.10	110		

Table S9. Expected and experimental fluorescence values and % signal recovery for biotin, N-acetyl-L-cysteine, thiourea, and thiocarbamate tested 2 and 4 days post spiking. Values are mean ± SD, tested in three replicates. Fluorescence signals are arbitrary units (AU). FI: Fluorescence Intensity

Theoretical Pd (ppm) in solid API		12	60	12	60	300	60
Pd in assay solution (nM)		3.76	6.26	11.2	18.8	31.3	56.3
API (mg/mL) in spiked sample		0.33	0.11	1	0.33	0.11	1
2 days post spike	FI = 1202×Pd(nM)	4520	7525	13462	22598	37623	67673
Biotin	Observed FI	6563	11678	22106	29517	37092	60188
	SD	655	2016	2345	2188	10251	4434
	% Signal recovery	145 ± 15	155 ± 27	164 ± 17	131 ± 10	99 ± 27	89 ± 7
N-Acetyl-cys	Observed FI	5088	9274	13712	26009	38040	47177
	SD	626	521	98	2038	1957	3015
	% Signal recovery	113 ± 14	123 ± 7	102 ± 1	115 ± 9	101 ± 5	70 ± 4
Thiourea	Observed FI	0	0	0	2556	11522	0
	SD				839	276	
	% Signal recovery	0	0	0	11 ± 4	31 ± 1	0
Thiocarbamate	Observed FI	5852	7560	19883	15527	32253	39174
	SD	793	276	2264	2124	3752	437
	% Signal recovery	129 ± 18	100 ± 4	148 ± 17	69 ± 9	86 ± 10	58 ± 1
4 days post spike	FI = 1317×Pd(nM)	4952	8244	14750	24760	41222	74147
Biotin	Observed FI	3409	10229	18113	26136	31848	48557
	SD	108	895	2540	921	2004	3154
	% Signal recovery	69 ± 2	124 ± 11	123 ± 17	106 ± 4	77 ± 5	65 ± 4
N-Acetyl-cys	Observed FI	2061	7654	6395	19360	32122	36602
	SD	321	387	119	424	1163	1592
	% Signal recovery	42 ± 6	93 ± 5	43 ± 1	78 ± 2	78 ± 3	49 ± 2
Thiourea	Observed FI	0	2437	0	2424	15045	880
	SD		638		371	2542	512
	% Signal recovery	0	30 ± 8	0	10 ± 1	36 ± 6	1 ± 1
Thiocarbamate	Observed FI	4613	3092	14052	6010	14577	15549
	SD	505	447	562	1311	1551	618
	% Signal recovery	93 ± 10	38 ± 5	95 ± 4	24 ± 5	35 ± 4	21 ± 1

Table S10. Expected and experimental fluorescence values and % signal recovery for penicillin tested 2 and 4 days post spiking.

Theoretical Pd (ppm) in solid API		12	60	12	60	300	60
Pd in assay solution (nM)		3.76	6.26	11.2	18.8	31.3	56.3
API (mg/mL) in spiked sample		0.33	0.11	1	0.33	0.11	1
2 days post spike	FI = 1099×Pd(nM)	4132	6880	12309	20661	34399	61874
Penicillin	Observed FI	4422	8446	7891	24237	35718	52353
	SD	340	530	222	923	3000	3671
	% Signal recovery	107 ± 8	123 ± 8	64 ± 2	117 ± 4	104 ± 9	85 ± 6
4 days post spike	FI = 1257×Pd(nM)	4726	7869	14078	23632	39344	70769
Penicillin	Observed FI	3708	8737	9176	21013	33725	43010
	SD	302	1124	1146	1769	2407	2485
	% Signal recovery	78 ± 6	111 ± 14	65 ± 8	89 ± 7	86 ± 6	61 ± 4

Detecting resin-bound palladium (Figure 3)

Table S11. Raw fluorescence values for detecting resin-bound palladium with or without the addition of $NaBH_4$.

Incubation time (h)	t_0	t_{30}	t_{60}	t_{18h}
Thiourea-based (+) $NaBH_4$	2470	205000	209000	223000
Thiol-based (+) $NaBH_4$	2560	203000	208000	207000
Thiourea-based (–) $NaBH_4$	1880	5070	17900	188000
Thiol-based (–) $NaBH_4$	1870	4830	26100	191000

Comparison of palladium standard solution, *t*BuXPhos-Pd-G3, and PEPPSI^{TM}-IPr for their catalytic activity (Figure 4)

Table S12. Raw fluorescence values of palladium standard solution, tBuXPhos-Pd-G3 and PEPPSI^{TM}-IPr (Figure 4b).

Pd Source:	Standard Solution		*t*BuXPhos-Pd-G3		PEPPSI^{TM}-IPr	
Pd (nM)	t_0	t_{15}	t_0	t_{15}	t_0	t_{15}
0	6812	28924	8258	32421	8854	31661
	6220	24302	8118	28074	7223	26321
	7528	30505	7961	29545	7481	28299
5	8215	43108	10708	49777	11574	49393
	9459	47610	8543	36335	9368	41344
	7754	35606	8544	34473	9565	40091
50	19788	99479	13197	85011	21587	100000
	18047	95004	14040	84356	24958	112290
	16897	103469	12878	80160	22548	98615

Table S13. Raw fluorescence values of palladium standard solution, tBuXPhos-Pd-G3 and PEPPSI^{TM}-IPr without addition of $NaBH_4$ (Figure 4c).

Pd Source:	Standard Solution		*t*BuXPhos-Pd-G3		PEPPSI^{TM}-IPr	
Pd (nM)	t_0	t_{15}	t_0	t_{15}	t_0	t_{15}
0	7329	7746	6905	6960	6808	6787
	7220	7522	6915	7007	6676	6398
	7109	7522	6818	6163	6822	6516
5	7080	6484	7078	6911	6823	6175
	7038	6507	6955	6220	6812	6213
	7007	6596	6715	5952	6827	6052
50	7121	6829	6962	6273	6719	6040
	7065	6812	7078	6394	7011	6355
	7294	6949	6857	5990	6957	6418

Quantification of palladium by standard addition method (Table 2)

Table S14a. Raw fluorescence values for quantification of palladium by method of standard addition for trials 1-3 for 80 ppb sample and trails 1 and 2 for 20, 5, and 0.2 ppb samples of solid-state palladium in ibuprofen. Fluorescence intensities at 0 min following addition of $NaBH_4$ were subtracted from fluorescence intensities following incubation for 30 min.

added Pd (nM)	0			210			420			840		
80 ppb palladium in solid state												
Trial	t_0	t_{30}	t_{30} - t_0	t_0	t_{30}	t_{30} - t_0	t_0	t_{30}	t_{30} - t_0	t_0	t_{30}	t_{30} - t_0
1	4130	6248	2118	4906	11980	7074	5926	17417	11491	7075	27056	19981
	4005	6711	2706	4889	15350	10461	5679	23312	17633	8267	47224	38957
	4190	7050	2860	4848	18090	13242	5805	29902	24097	6701	55935	49234
2	4279	11277	6998	4794	28609	23815	5850	51122	45271	7778	82880	75102
	4216	9474	5257	5460	27844	22384	7193	45841	38648	9666	93484	83818
	4402	9761	5359	5385	26981	21596	7247	47297	40050	10182	87421	77239
3	4067	8889	4822	5147	18066	12919	7169	44539	37370	8334	50175	41841
	4185	7503	3318	4879	15822	10943	6500	28369	21869	9229	53937	44708
	4304	9250	4946	4557	14323	9766	5841	29610	23769	8631	49604	40973
20 ppb palladium in solid state												
Trial	t_0	t_{30}	t_{30} - t_0	t_0	t_{30}	t_{30} - t_0	t_0	t_{30}	t_{30} - t_0	t_0	t_{30}	t_{30} - t_0
1	4337	7851	3514	6098	24242	18144	10352	84383	74031	12679	80264	67585
	4361	7747	3386	6697	25659	18961	8717	48367	39650	12592	81758	69166
	4465	7223	2759	7166	22812	15646	10867	50237	39369	16674	85458	68785
2	3755	6327	2572	4461	13441	8980	5147	39059	33912	7357	51643	44286
	3706	5553	1847	4628	15276	10648	6144	32001	25857	7489	51160	43671
	4513	8022	3509	5455	18582	13127	5851	29545	23694	8769	50916	42147
5 ppb palladium in solid state												
Trial	t_0	t_{30}	t_{30} - t_0	t_0	t_{30}	t_{30} - t_0	t_0	t_{30}	t_{30} - t_0	t_0	t_{30}	t_{30} - t_0
1	4133	10674	6541	4850	27259	22408	6079	51535	45456	7961	78397	70436
	4130	9324	5194	5526	30372	24846	7032	52136	45104	10996	90760	79765
	4363	9837	5474	4486	26765	22279	7804	51410	43606	10231	77698	67468
2	3760	6578	2818	4882	20741	15859	5797	32694	26897	8889	53114	44225
	3915	6679	2764	4656	16797	12141	6288	33019	26731	8019	47500	39481
	3723	5856	2133	4364	17227	12863	5812	26855	21043	9204	54695	45491
0.2 ppb palladium in solid state												
Trial	t_0	t_{30}	t_{30} - t_0	t_0	t_{30}	t_{30} - t_0	t_0	t_{30}	t_{30} - t_0	t_0	t_{30}	t_{30} - t_0
1	4289	10213	5924	6166	28060	21895	8502	48893	40391	12708	94642	81934
	4352	9851	5499	6376	31447	25071	9012	49426	40414	14410	83590	69181
	4423	9721	5297	6840	29695	22855	9939	47590	37651	15643	86707	71064
2	3549	6469	2920	4275	16806	12531	4863	30258	25395	6521	54320	47799
	3737	6471	2734	4511	18607	14096	6346	38715	32369	8135	63447	55312
	4149	7454	3305	5099	18398	13299	7789	36575	28786	9304	74184	64880

Table S14b. Raw fluorescence values for quantification of palladium by method of standard addition for trials 4 and 5 for 80 ppb sample and trials 3 and 4 for 20, 5, and 0.2 ppb samples of solid-state palladium in ibuprofen. Fluorescence intensities of wells without $NaBH_4$ (reaction blanks) were subtracted from fluorescence intensities of wells containing $NaBH_4$ following incubation for 30 min.

added Pd (nM)	0			210			420			840		
80 ppb palladium in solid state												
Trial	t_{30}	$t_{30\ blank}$	t_{30}- $t_{30\ blank}$	t_{30}	$t_{30\ blank}$	t_{30}- $t_{30\ blank}$	t_{30}	$t_{30\ blank}$	t_{30}- $t_{30\ blank}$	t_{30}	$t_{30\ blank}$	t_{30}- $t_{30\ blank}$
4	11276	4035	7241	26091	3909	22181	37355	3515	33840	70007	3630	66377
	10616		6581	27389		23480	49993		46478	80937		77307
5	11244	4224	7020	26464	3731	22733	55126	3884	51242	83392	4318	79074
	11720		7496	32279		28548	52076		48192	96199		91881
20 ppb palladium in solid state												
Trial	t_{30}	$t_{30\ blank}$	t_{30}- $t_{30\ blank}$	t_{30}	$t_{30\ blank}$	t_{30}- $t_{30\ blank}$	t_{30}	$t_{30\ blank}$	t_{30}- $t_{30\ blank}$	t_{30}	$t_{30\ blank}$	t_{30}- $t_{30\ blank}$
3	8446	4115	4331	25164	3611	21553	48587	3487	45100	79381	3598	75783
	7901		3786	24681		21070	40404		36917	79843		76244
4	8329	4163	4166	23350	3770	19580	44872	3755	41117	83099	3775	79324
	8790		4627	25615		21845	47052		43297	86812		83037
5 ppb palladium in solid state												
Trial	t_{30}	$t_{30\ blank}$	t_{30}- $t_{30\ blank}$	t_{30}	$t_{30\ blank}$	t_{30}- $t_{30\ blank}$	t_{30}	$t_{30\ blank}$	t_{30}- $t_{30\ blank}$	t_{30}	$t_{30\ blank}$	t_{30}- $t_{30\ blank}$
3	8116	3699	4416	24135	3788	20347	38219	3707	34511	75302	3732	71570
	8538		4838	26583		22795	42800		39093	77395		73663
4	9010	3682	5328	24164	3876	20288	45490	3793	41697	87325	3738	83587
	8403		4721	31739		27863	48327		44534	81384		77646
0.2 ppb palladium in solid state												
Trial	t_{30}	$t_{30\ blank}$	t_{30}- $t_{30\ blank}$	t_{30}	$t_{30\ blank}$	t_{30}- $t_{30\ blank}$	t_{30}	$t_{30\ blank}$	t_{30}- $t_{30\ blank}$	t_{30}	$t_{30\ blank}$	t_{30}- $t_{30\ blank}$
3	9118	3663	5455	23460	3651	19809	40209	3649	36560	71474	3572	67902
	8605		4942	25188		21537	43319		39670	79081		75509
4	8433	3676	4757	27864	3716	24148	47649	3721	43928	82341	3593	78748
	8291		4615	28681		24965	47767		44046	77955		74362

Table S15. Linear-regression equations and extrapolated x-intercepts used to back-calculate palladium content in various spiked samples. Reported measurements are expressed as the average of these values.

Pd (ppb)	Trial 1	Trial 2	Trial 3	Trial 4	Trial 5
80	838.7×X + 1977	1831×X + 5083	988.8×X + 4133	1629×X + 6911	1975×X + 7459
|x-intercept|	2.357	2.776	4.18	4.242	3.776
20	*	1692×X + 5466	1048×X + 2844	1805×X + 4059	1943×X + 3129
|x-intercept|		3.23	2.713	2.249	1.611
5	*	1680×X + 7146	1010×X + 3356	1682×X + 4627	1889×X + 5145
|x-intercept|		4.253	3.322	2.751	2.723
0.2	*	1706×X + 5739	1349×X + 1686	1654×X + 5199	179×X + 6120
|x-intercept|		3.363	1.25	3.143	3.419

*not tested

|x-intercept| = [Pd] (nM) in wells

Back calculation

$$\frac{x\ \text{nmol Pd}}{\text{L}} \times \frac{210\ \mu\text{L}}{20\ \mu\text{L}} \times \frac{1\ \text{L}}{1000\ \text{mL}} \times \frac{106.42\ \text{ng Pd}}{\text{nmol Pd}} \times \frac{1\ \text{mL}}{40\ \text{mg ibu}} = \frac{y\ \text{ng Pd}}{\text{mg ibu}} = y\ \text{ppm} = 1000y\ \text{ppb}$$

a

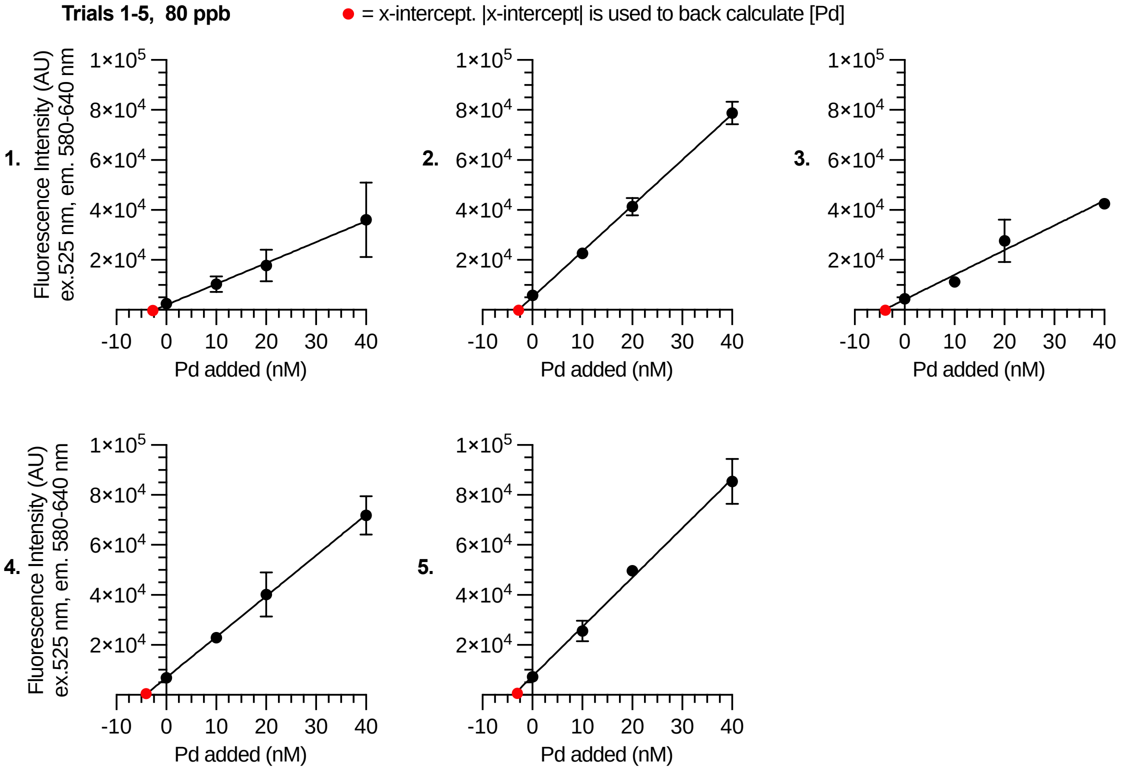

b

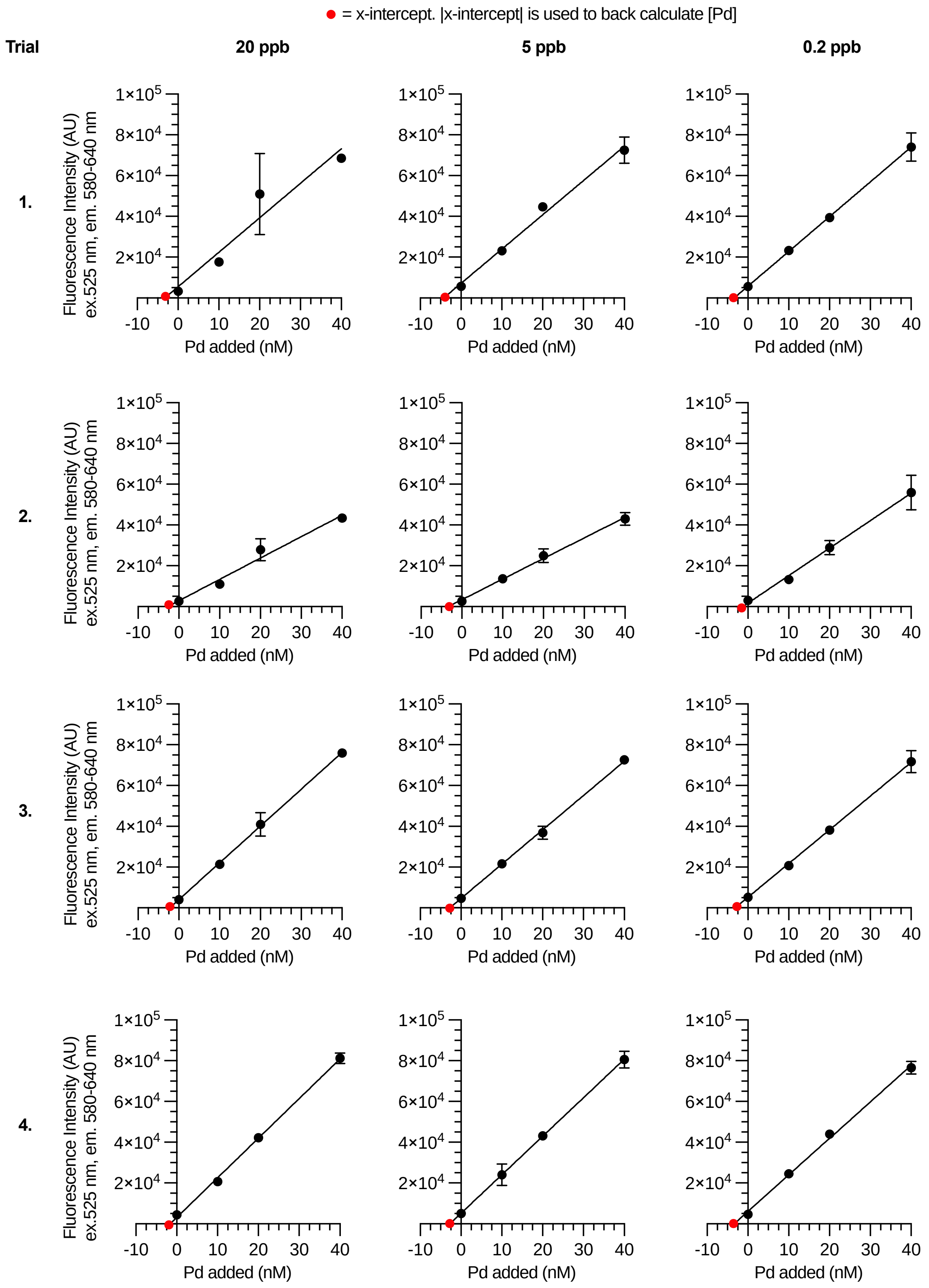

Figure S3. Standard addition curves for palladium-spiked ibuprofen samples: 80 (a), 20, 5, and 0.2 (b) ppb. Each linear-regression line was extrapolated to the x-intercept to calculate experimental [palladium]. Reported measurements are expressed as the average of these values.

Investigation of compatibility with palladium nanoparticles (Figure 5)

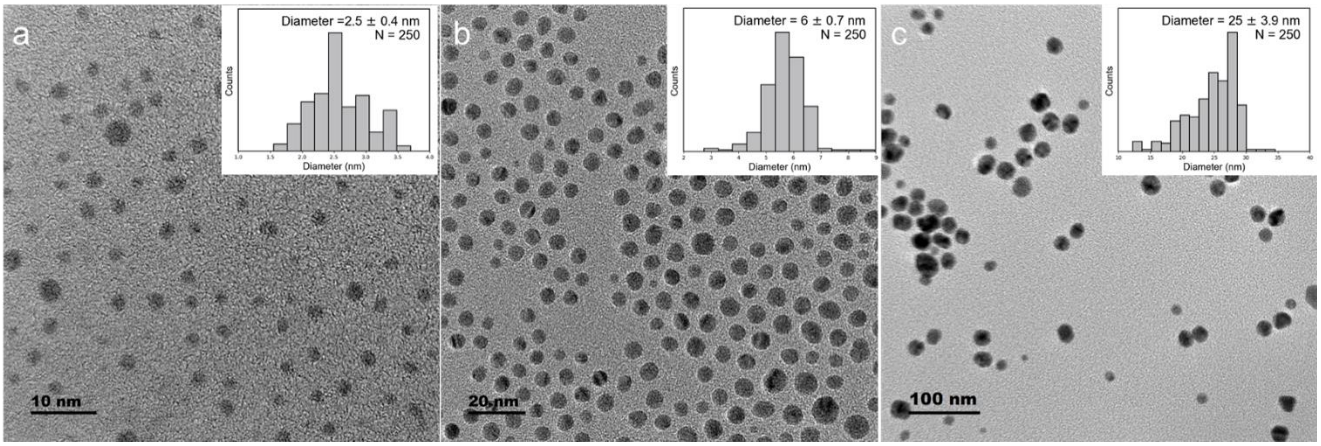

Figure S4. Representative bright field transmission electron microscopy images and size histograms for 2 nm (a), 5 nm (b), and 25 nm (c) Pd NPs after functionalization with 1kDa PEGSH.

Table S16. Raw fluorescence values for palladium standard solutions and 0 nM Pd with and without acid.

0 nM Pd									
Condition:	t_0			t_{15}			t_{15}-t_0		
water	9394	8789	8694	11488	11677	11350	2094	2888	2656
5% aqua regia	8797	8346	8630	11607	10237	10815	2810	1891	2185
20% aqua regia	9541	8127	8168	12928	10000	9452	3387	1873	1284
**50 nM Pd**									
Condition:	t_0			t_{15}			t_{15}-t_0		
water	73877	85917	71821	124072	129584	129175	50195	43667	57354
5% aqua regia	71824	70475	69216	121322	120128	127167	49498	49653	57951
20% aqua regia	73148	86966	81919	135729	138198	151657	62582	51232	69738

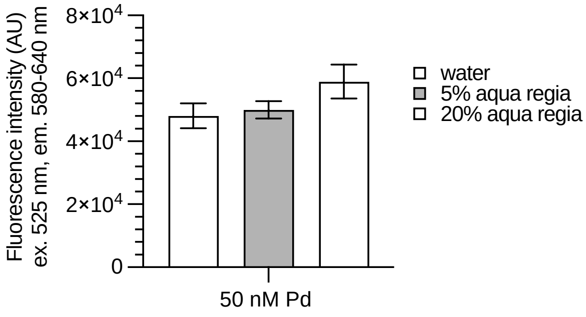

Figure S5. Figure illustrating acid digestion with 5% and 20% aqua regia does not affect assay and fluorescence output. Fluorescence intensities measured immediately after $NaBH_4$ addition (t_0) were subtracted from intensities measured after 15 minutes. Remaining fluorescence of 0 nM palladium samples were averaged and subtracted from 50 nM palladium samples.

Table S17a. Raw fluorescence values for palladium nanoparticles (2 nm, 5 nm, and 25 nm) without acid digestion. Palladium concentration is 50 nM based on dilution from 1.0 mM palladium in Pd NPs determined by ICP-OES.

Pd NP (nm)					2				
Pd (nM)		t_0			t_{15}			t_{15}-t_0	
25	8583	8645	8615	15176	14519	14416	6594	5874	5802
50	9264	8621	9465	16650	15994	16238	7386	7373	6773
100	9845	10108	10121	19511	21053	20490	9666	10945	10370
Pd NP (nm)					5				
Pd (nM)		t_0			t_{15}			t_{15}-t_0	
25	8307	8487	8889	15517	14607	15492	7210	6119	6603
50	9227	8637	9372	17855	17063	17334	8629	8425	7962
100	10521	10602	10620	23657	25613	24832	13135	15011	14212
Pd NP (nm)					25				
Pd (nM)		t_0			t_{15}			t_{15}-t_0	
25	8820	8778	9096	16903	16603	18480	8083	7825	9384
50	9433	8921	9857	22150	21353	21787	12717	12432	11929
100	11485	11639	11672	29894	33501	33600	18409	21862	21928

Table S17b. Raw fluorescence values for palladium nanoparticles (2 nm, 5 nm, and 25 nm) acid digested with 20% aqua regia. Palladium concentration is 50 nM based on dilution from 1.0 mM palladium in Pd NPs determined by ICP-OES.

Pd NP (nm)					2				
Pd (nM)		t_0			t_{15}			t_{15}-t_0	
25	9001	8812	8881	17979	17685	18763	8978	8873	9883
50	9620	9118	10125	25169	24695	24493	15548	15577	14368
100	11686	12393	11667	35371	40042	39170	23684	27649	27503
Pd NP (nm)					5				
Pd (nM)		t_0			t_{15}			t_{15}-t_0	
25	9260	9486	9759	20818	20721	23232	11558	11235	13474
50	11226	10465	11530	32934	31974	31264	21709	21509	19734
100	12876	15786	14550	42725	54170	52011	29849	38384	37461
Pd NP (nm)					25				
Pd (nM)		t_0			t_{15}			t_{15}-t_0	
25	10345	10490	11082	25701	28984	30442	15356	18494	19360
50	13879	12595	14138	48958	47987	47519	35079	35392	33382
100	18689	23402	20875	76325	87129	81926	57636	63727	61052

Table S17c. Raw fluorescence values for 0 nM palladium control. Remaining fluorescence of the 0 nM palladium solution was averaged and subtracted from values in Tables S17a and S17b so that intercept of Figure 5 is set at the origin and analysis is consistent with Table 1 and Figure S5.

	0 nM Pd		
t_0	9062	8393	7966
t_{15}	12701	12563	12446
t_{15}-t_0	3639	4170	4480

Cited References

1. Koide, K.; Tracey, M. P.; Bu, X.; Jo, J.; Williams, M. J.; Welch, C. J., A competitive and reversible deactivation approach to catalysis-based quantitative assays. *Nat. Commun.* **2016,** *7*, 10691.

2. E, X.; Zhang, Y.; Zou, J.-J.; Wang, L.; Zhang, X. Oleylamine-protected metal (Pt, Pd) nanoparticles for pseudohomogeneous catalytic cracking of JP-10 jet fuel. *Ind. Eng. Chem. Res.* **2014**, *53*, 12312–12318.

3. Mazumder, V.; Sun, S. Oleylamine-mediated synthesis of Pd nanoparticles for catalytic formic acid oxidation. *J. Am. Chem. Soc.* **2009**, *131*, 4588–4589.
